# Reply to ‘Sacubitril/valsartan in heart failure: a critical look at dementia risk and future study pathways’

**DOI:** 10.1093/ehjcvp/pvaf082

**Published:** 2025-11-16

**Authors:** Kyungyeon Jung, Ju-Young Shin, Ju Hwan Kim

**Affiliations:** Department of Biohealth Regulatory Science, Sungkyunkwan University, 2066 Seobu-ro, Jangan-gu, Suwon-si, Gyeonggi-do 16419, South Korea; Department of Biohealth Regulatory Science, Sungkyunkwan University, 2066 Seobu-ro, Jangan-gu, Suwon-si, Gyeonggi-do 16419, South Korea; School of Pharmacy, Sungkyunkwan University, 2066 Seobu-ro, Jangan-gu, Suwon-si, Gyeonggi-do 16419, South Korea; Department of Clinical Research Design & Evaluation, Samsung Advanced Institute for Health Sciences & Technology, Sungkyunkwan University, Seoul 06355, South Korea; Department of Biohealth Regulatory Science, Sungkyunkwan University, 2066 Seobu-ro, Jangan-gu, Suwon-si, Gyeonggi-do 16419, South Korea; School of Pharmacy, Sungkyunkwan University, 2066 Seobu-ro, Jangan-gu, Suwon-si, Gyeonggi-do 16419, South Korea

We would like to thank Dong and colleagues for their thoughtful comments^1^ regarding our recent study on the association between sacubitril/valsartan use and the risk of dementia in patients with heart failure.^2^ Their remarks provide an opportunity to further clarify the interpretation of our findings and may help guide future research.

First, Dong *et al.* highlighted the potential for residual confounding inherent to observational studies using claims data, which may not be fully addressed despite adjustment for a wide range of covariates. Indeed, key clinical parameters such as estimated glomerular filtration rate, left ventricular ejection fraction (LVEF), and N-terminal pro–B-type natriuretic peptide—important predictors of dementia—were not available in our database,^3-5^ and this limitation should be acknowledged when interpreting our results. As thoroughly discussed in our paper, an exploratory supplementary analysis utilizing electronic medical record data from a single institution showed that patients in the sacubitril/valsartan group were more likely to have lower LVEF than those in the comparator group, with 92.0% and 46.1% of patients having LVEF <40%, respectively. Although reduced LVEF has been linked to a higher risk of cognitive impairment and dementia,^5^ improved cardiac function with sacubitril/valsartan may counteract the potential cognitive effects previously attributed to sacubitril. Further studies incorporating quantitative bias analysis may help contextualize and interpret these findings.

Second, although we used a previously validated claims-based definition of dementia, our study was limited to identifying clinically diagnosed cases based on diagnosis and prescription records. Etiologic diagnosis of dementia, such as Alzheimer’s disease, can be established using the AT(N) biomarker framework.^6^ However, this approach could not be applied in our study because data on fluid-based or imaging biomarkers were not available in the claims database. In addition, the clinical severity of cognitive decline could not be assessed, as such evaluations typically require prospective data, and functional measures such as the Mini-Mental State Examination were not available in our data source. As suggested by Dong *et al.*, future studies incorporating standardized neuropsychological assessments and neuroimaging modalities would enable more accurate detection of early cognitive changes and differentiation between dementia subtypes. Moreover, the relatively short mean follow-up period in our study may have limited our ability to capture long-term dementia outcomes.

Third, as Dong *et al*. noted, our study included a high proportion of male patients, consistent with previous studies.^7,8^ This observation warrants caution when interpreting the results, as sex differences exist in both dementia risk and subtype,^9,10^ and our subgroup analysis also indicated a higher incidence of composite dementia among female patients. While our findings reflect real-world prescribing patterns of sacubitril/valsartan in South Korea, further sex-specific analyses would be valuable.

Lastly, as Dong *et al*. suggested, plausible vascular mechanisms—such as improvements in left ventricular remodelling in heart failure—may support a cardio-cerebral link,^11^ which could explain the observed hazard ratio below 1 for overall dementia risk. However, these findings should be interpreted with caution, as the most dementia cases in our study were identified using diagnosis and prescription codes for Alzheimer’s dementia. Further studies exploring the risk of specific dementia subtypes and their underlying mechanisms are warranted.

We appreciate the constructive feedback from Dong and colleagues, which provides important context for interpreting our findings and may inform future research and clinical decision-making regarding the potential neurocognitive effects of sacubitril/valsartan in patients with heart failure.

## Data Availability

There are no new data associated with this article.
